# Non-Specific Low Back Pain and Lumbar Radiculopathy: Comparison of Morphologic and Compositional MRI as Assessed by gagCEST Imaging at 3T

**DOI:** 10.3390/diagnostics11030402

**Published:** 2021-02-26

**Authors:** Miriam Frenken, Sven Nebelung, Christoph Schleich, Anja Müller-Lutz, Karl Ludger Radke, Benedikt Kamp, Matthias Boschheidgen, Lena Wollschläger, Bernd Bittersohl, Gerald Antoch, Markus R. Konieczny, Daniel B. Abrar

**Affiliations:** 1 Department of Diagnostic and Interventional Radiology, Medical Faculty, University Dusseldorf, D-40225 Dusseldorf, Germany; Miriam.Frenken@med.uni-duesseldorf.de (M.F.); sven.nebelung@med.uni-duesseldorf.de (S.N.); Christoph.Schleich@med.uni-duesseldorf.de (C.S.); Anja.Lutz@med.uni-duesseldorf.de (A.M.-L.); Ludger.Radke@med.uni-duesseldorf.de (K.L.R.); Benedikt.Kamp@med.uni-duesseldorf.de (B.K.); Matthias.Boschheidgen@med.uni-duesseldorf.de (M.B.); Antoch@med.uni-duesseldorf.de (G.A.); 2 Department of Orthopedic and Trauma Surgery, University Hospital of Duesseldorf, D-40225 Duesseldorf, Germany; Lena.wollschlaeger@med.uni-duesseldorf.de (L.W.); Bernd.Bittersohl@med.uni-duesseldorf.de (B.B.); Markus.Konieczny@med.uni-duesseldorf.de (M.R.K.)

**Keywords:** gagCEST, spine, compositional MRI, disk degeneration, low back pain, radiculopathy, disk extrusion, IVD

## Abstract

Using glycosaminoglycan Chemical Exchange Saturation Transfer (gagCEST) magnetic resonance imaging (MRI), this study comparatively evaluated the GAG contents of lumbar intervertebral disks (IVDs) of patients with non-specific low back pain (nsLBP), radiculopathy, and asymptomatic volunteers to elucidate the association of clinical manifestation and compositional correlate. A total of 18 patients (mean age 57.5 ± 22.5 years) with radiculopathy, 16 age-matched patients with chronic nsLBP and 20 age-matched volunteers underwent standard morphologic and compositional gagCEST MRI on a 3T scanner. In all cohorts, GAG contents of lumbar IVDs were determined using gagCEST MRI. An assessment of morphologic IVD degeneration based on the Pfirrmann classification and T2-weighted sequences served as a reference. A linear mixed model adjusted for multiple confounders was used for statistical evaluation. IVDs of patients with nsLBP showed lower gagCEST values than those of volunteers (nsLBP: 1.3% [99% confidence intervals (CI): 1.0; 1.6] vs. volunteers: 1.9% [99% CI: 1.6; 2.2]). Yet, IVDs of patients with radiculopathy (1.8% [99% CI: 1.4; 2.1]) were not different from patients with nsLBP or volunteers. In patients with radiculopathy, IVDs directly adjacent to IVD extrusions demonstrated lower gagCEST values than distant IVDs (adjacent: 0.9% [99% CI: 0.3; 1.5], distant: 2.1% [99% CI: 1.7; 2.5]). Advanced GAG depletion in nsLBP and directly adjacent to IVD extrusions in radiculopathy indicates close interrelatedness of clinical pathology and compositional degeneration.

## 1. Introduction

Low back pain (LBP) is a major global health burden that is associated with limited physical activity, increased disability, and absence from work [1]. Therefore, LBP is characterized by an enormous individual and socioeconomic disease burden. The majority of LBP is non-specific (nsLBP), i.e., without an unequivocal structural cause such as vertebral fractures [2,3]. One potential contributor to nsLBP is lumbar degenerative disk disease (LDDD), which is an accelerated type of age-related intervertebral disk (IVD) degeneration [4,5]. A very common structural disorder leading to LBP is lumbar IVD extrusion with radiculopathy [6,7,8]. To this date, the importance of magnetic resonance imaging (MRI) at LBP is still controversial, especially in the acute and subacute setting, and therefore, it is not recommended for primary diagnostics by current guidelines [9,10]. However, it is the most widely used imaging technique for the direct assessment of the characteristic morphologic changes in LDDD [11,12], such as IVD dehydration and loss of IVD height. In the clinical routine, these changes are commonly visualized by T2-weighted (T2w) images and allow for the differentiation of degenerated and non-degenerated IVDs according to validated grading systems such as the Pfirrmann classification [13,14]. However, these standard clinical MRI techniques allow assessment of mere morphology and cannot depict early, potentially reversible changes of IVD composition such as glycosaminoglycan (GAG) depletion that precede structural changes. Consequently, compositional MRI techniques such as GAG Chemical Exchange Saturation Transfer (gagCEST) imaging that evaluate tissue properties beyond morphology are of ever-increasing clinical and scientific interest [15,16,17,18,19]. As a non-invasive imaging technique, gagCEST measures the chemical exchange of hydroxy protons between GAG and bulk water molecules [20,21,22]. To induce the CEST effect in the tissue, a frequency-specific radiofrequency (RF) pulse is used to saturate a pool of solute protons at different frequency offsets around the water resonance. These saturated protons are subsequently transferred to the bulk water pool by chemical exchange, consequently reducing its signal. The signal decrease is then used to assess the CEST effect at a GAG-specific frequency range of 0.9–1.9 ppm [22,23]. The resulting magnetization transfer ratio asymmetry (MTR_asym_), i.e., the gagCEST effect size, correlates with the underlying GAG concentration of the given IVD [23,24]. In previous studies, our group has provided preliminary evidence that gagCEST imaging of the lumbar spine may help to differentiate degenerative and non-degenerative IVDs based on their respective GAG contents [24,25,26]. Yet, the association of compositional changes, i.e., GAG depletion, and common clinical manifestations of disorders of the lumbar spine remains to be elucidated.

Against this background, the aim of this study was to systematically assess the GAG contents of lumbar IVDs in common disorders of the lumbar spine, i.e., nsLBP and radiculopathy, and to compare them to asymptomatic volunteers, both overall and on a segmental level and to elucidate a potential influence of IVD extrusions on the GAG contents of adjacent IVDs. We hypothesized that (a) the GAG contents in patients with nsLBP and radiculopathy are significantly lower than in asymptomatic volunteers and that (b) the GAG contents of lumbar IVDs adjacent to extruded IVDs is significantly lower than of non-adjacent IVDs.

## 2. Materials and Methods

### 2.1. Study Population

A total of 18 patients (mean age: 57.5 ± 22.5 years, range: 14–96 years; 10 female, 8 male) with subacute (4–12 weeks duration) [25] radiculopathy and IVD extrusion and 16 age-matched patients with chronic nsLBP (mean age: 59.0 ± 17.5 years, range: 22.5–83.5 years; 10 female, 6 male) were prospectively recruited at the outpatient clinic of the Department of Orthopedic and Trauma Surgery of the University Hospital Duesseldorf. In patients with radiculopathy, IVD extrusion had been diagnosed earlier during previous MRI scanning sessions and was based on the recommendations of the combined task forces of the North American Spine Society, American Society of Spine Radiology, and American Society of Neuroradiology [26]. Accordingly, extrusion was defined as being present when any distance between the edges of the disc material beyond the disc space was greater than the distance between the edges of the base. Additionally, 20 age-matched asymptomatic volunteers (mean age: 54.5 ± 11.5 years, range: 39.5–76.5 years; 11 female, 9 male) were included as a control group. Chronic LBP was defined as persistent LBP symptoms beyond three months [27]. Exclusion criteria for all participants were prior spine surgery, chronic inflammatory diseases affecting the musculoskeletal system, congenital spine deformities, and a body mass index < 18.5 or >30 kg/m^2^. For the control group, the exclusion criteria were expanded to acquired spinal deformities, radiculopathy, and chronic LBP. For the LBP group, the exclusion criteria were expanded to presence of radiculopathy. All patients were treated conservatively. Written and informed consent was obtained from all participants or their legal guardians prior to the initiation of the study. The study was approved by the local ethical committee (Ethical Committee, Medical Faculty, University of Düsseldorf, Germany, study number 5087R, 29 June 2015). Demographic and clinical characteristics of the study population and the three study cohorts are presented in Table 1.

### 2.2. Clinical Assessment

Patients were clinically assessed at the outpatient clinic of the Department of Orthopedic and Trauma Surgery by a board-certified orthopedic surgeon (MRK, 10 years of experience as a spine surgeon). Patients and volunteers underwent a focused neurologic exam of the motor and sensory systems as well as the reflex status. More specifically, back and leg pain with a particular focus on radicular pain, distal sensation and muscle strength were evaluated. Wherever radicular pain, i.e., pain in a nerve-root distribution, sensory loss or muscle weakness were present, the level of nerve affection was clinically localized using the corresponding dermatoma or myotoma.

### 2.3. Imaging

MRI studies of the lumbar spines of all participants were performed on a clinical 3 Tesla (T) MRI scanner (Magnetom Prisma, Siemens Healthineers, Erlangen, Germany) with a 32-channel body and a 24-channel spine matrix coil (Siemens Healthineers, Erlangen, Germany) in the supine position.

In line with earlier studies, the MRI exams comprised both morphologic and compositional sequences. The morphologic sequences included T1-weighted (T1w), T2-weighted (T2w), and short-tau-inversion-recovery (STIR) sequences in the sagittal orientation and a T2w sequence in the transversal orientation. Compositional imaging included CEST and Water Saturation Shift Referencing (WASSR) sequences to compensate for magnetic field inhomogeneities in the sagittal orientation. Detailed sequence parameters are presented in Table 2.

### 2.4. Image Analysis

The raters were blinded to the participants’ diagnoses and demographics. The lumbar IVDs (segments L1/L2–L5/S1) of all participants were graded individually and independently on sagittal T2w images according to the Pfirrmann classification by two radiologists with long-standing experience in musculoskeletal imaging (DBA and CS with four and ten years of experience in musculoskeletal imaging) [11]. In case of diverging findings, consensus was reached with assistance of a third clinical radiologist (SN, eight years of experience in musculoskeletal imaging). The Pfirrmann classification allows distinction of non-degenerative (grades 1 and 2) and degenerative IVDs (grades 3–5) on the basis of signal intensity, structure, and height as well as the distinction of the nucleus pulposus (NP) and annulus fibrosus (AF).

In addition, all images and all lumbar segments were analyzed by the same raters for the presence of IVD extrusions according to the recommendations of the Combined Task Forces of the North American Spine Society, American Society of Spine Radiology, and American Society of Neuroradiology [26]. Consequently, extrusion was defined as being present when any distance between the edges of the disc material beyond the disc space was greater than the distance between the edges of the base. As published in previous studies [16,28], we first used WASSR images to correct B_0_ field inhomogeneities by the maximum-symmetry algorithm with calculation of a pixel-wise frequency offset curve. Second, the corrected CEST curves divided by the signal without pre-saturation (S_0_) were defined as the so-called z-spectra (Z (ω)). The maximum frequency offset of each z-spectrum was Δω = 3 ppm. MTR_asym_ (defined as MTR_asym_(Δω) = Z(−Δω)−Z(Δω)) was used for the assessment of the gagCEST effect [16]. We calculated MTR_asym_ maps by using the average value of MTR_asym_ in the GAG-specific frequency range of Δω = 0.9–1.9 ppm. MTR_asym_ values, i.e., the gagCEST effect sizes, are given in [%] [29]. As previously published by our group, regions of interest (ROIs) were defined in the midsagittal plane by a customized in-house script implemented in Matlab (MATLAB, R2018a, The MathWorks, Inc., MA, USA) that automatically identifies lumbar segments and analyzes the gagCEST effect [30].

The disk segmentation approach was based on Bayes classification to divide bone and ligaments from disk cartilage [31]. All ROIs were visually confirmed for correct positioning by a board-certified radiologist (CS). No ROIs had to be manually corrected. In the following, MTR_asym_ values are referred to as gagCEST values for better readability. As shown in previous studies, gagCEST values correlate with the IVD’s GAG content [16,28]. Therefore, lower gagCEST values reflect lower GAG contents.

### 2.5. Statistical Analysis

SPSS software (v27, SPSS Inc., Chicago, IL, USA) was used for all statistical analyses performed by KLR and DBA. Descriptive statistics of gagCEST values were calculated for volunteers and patients. Based on a linear mixed model (LMM), the three study cohorts, i.e., asymptomatic volunteers, patients with nsLBP and patients with radiculopathy, IVD regions, i.e., NP and AF, and IVD segments, i.e., L1/2 to L5/S1 were comparatively evaluated as multivariable statistics. The model included a subject-specific random intercept, the factors healthy volunteer/patient, age, gender, Pfirrmann grading, and the interaction of these factors and was fitted using a restricted maximum likelihood approach [32]. Based on another LMM, IVDs adjacent to IVD extrusions, i.e., directly above or below, and non-adjacent IVDs were comparatively evaluated. The model included the factors adjacent/non-adjacent IVD, age, gender, Pfirrmann grading and the interaction of these factors. Based on these final models, the mean differences of gagCEST values were calculated. Not assuming normal distributions, mean Pfirrmann grades were compared between the three study cohorts using the Kruskal–Wallis test followed by Dunn’s post-hoc test wherever appropriate. The presentation of *p*-values for the illustration of statistical significance was deliberately avoided [33].

## 3. Results

### 3.1. Morphologic Analysis

Detailed findings of IVD extrusion and IVD degradation are given in Table 1 and Table 3, respectively. Only patients with radiculopathy were found to demonstrate IVD extrusions at the IVD segments L4/5 and L5/S1, while patients with nsLBP or asymptomatic volunteers showed none (Table 1).

The grading of IVDs according to the Pfirrmann classification was as follows: entire study population: grade 1: (*n* = 0), grade 2: (*n* = 192), grade 3: (*n* = 79), grade 4: (*n*= 29), and grade 5: (*n* = 2); patients with radiculopathy: grade 1: (*n* = 0), grade 2: (*n* = 48), grade 3: (*n* = 31), grade 4: (*n*= 11), and grade 5: (*n* = 0); patients with nsLBP: grade 1: (*n* = 0), grade 2: (*n* = 37), grade 3: (*n* = 30), grade 4: (*n*= 11), and grade 5: (*n* = 2); asymptomatic volunteers: grade 1: (*n* = 0), grade 2: (*n* = 70), grade 3: (*n* = 19), grade 4: (*n* = 7), and grade 5: (*n* = 0). At each individual lumbar segment, we did not find distinct differences for the Pfirrmann grades between the three study cohorts. However, when considering all lumbar segments, patients with nsLBP had lower overall Pfirrmann grades than asymptomatic volunteers (nsLBP: 2.7 [99% CI: 2.6; 2.9] vs. volunteers: 2.4 [99% CI: 2.2; 2.5]). No distinct differences in Pfirrmann grades were found between patients with nsLBP and patients with radiculopathy and between patients with radiculopathy and asymptomatic volunteers (Table 3).

### 3.2. Multivariable Comparative Analyses of gagCEST Values

In all IVDs, irrespective of the study cohort, the NPs showed higher gagCEST values than the AFs (AF: 1.2% [99% CI: 1.0; 1.4] vs. NP: 2.1% [99% CI 1.8; 2.4], *p* < 0.001). GagCEST values were affected by morphologic IVD degeneration as assessed by the Pfirrmann grade. Overall, IVDs with Pfirrmann grades ≤ 2, i.e., non-degenerated IVDs, had higher gagCEST values (2.0% [99% CI: 1.7; 2.2]) than IVDs with Pfirrmann grades ≥ 3, i.e., degenerated IVDs (1.3% [99% CI: 1.0; 1.6]) (*p* < 0.001). No distinct differences were found between gagCEST values of all IVDs of the different lumbar segments: L1/2: 1.9% [99% CI: 1.5; 2.3]; L2/3: 1.6% [99% CI: 1.2; 1.9]; L3/4: 1.5% [99% CI: 1.1; 1.8], L4/5: 1.4% [99% CI: 1.1; 1.7], L5/S1: 1.9% [99% CI: 1.4; 2.5].

**Figure 1:**
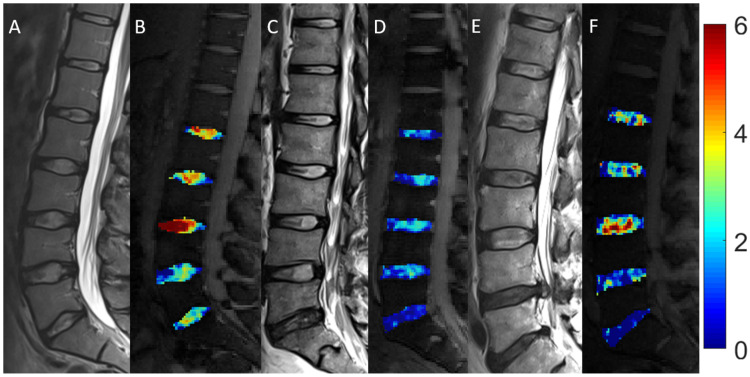
Morphologic and compositional imaging findings of lumbar intervertebral discs of an asymptomatic volunteer (**A**,**B**), a patient with nonspecific low back pain (**C**,**D**), and a patient with radiculopathy (**E**,**F**). A, C and E: Sagittal T2-weighted (T2w) images show the absence of morphologic signs of relevant IVD degeneration (**A**), substantial dehydration at the L4/L5 segment (**C**) and the L5/S1 segment (**C**,**E**) accompanied by extrusion at the L4/L5 segment (**E**). B, D and F: Sagittal glycosaminoglycan Chemical Exchange Saturation Transfer (gagCEST) images with overlaid color-coded maps to visualize the GAG contents of the IVD segments. Low GAG content is depicted in blue, and high GAG content is depicted in red. The unit of scale on the right is gagCEST effect in %. The lowest values are found in the patient with nsLBP, while the highest values are seen in the asymptomatic volunteer.

Comparative analyses of gagCEST values of lumbar IVDs in the three study cohorts are presented in Table 3 and Figure 1.

Overall, the lumbar IVDs of patients with nsLBP showed lower gagCEST values (1.3% [99% CI: 1.0; 1.6) than those of asymptomatic volunteers (1.9% [99% CI: 1.6; 2.2]) and lower values than those of patients with radiculopathy (1.8% [99% CI: 1.4; 2.1]). No distinct differences in gagCEST values were found between patients with radiculopathy and asymptomatic volunteers or between female (1.7% [99% CI: 1.5; 2.0]) and male individuals (1.6% [99% CI: 1.3; 1.8]) in the entire study population.

Comparative analyses of gagCEST values of IVDs adjacent and non-adjacent to IVD extrusions in patients with radiculopathy are displayed in Table 4.

IVDs adjacent to an IVD extrusion, i.e., directly above and/or below, demonstrated lower gagCEST values (0.9% [99% CI: 0.3; 1.5]) than IVDs that were non-adjacent, i.e., distant, to an extruded IVD (1.9% [99% CI: 1.7; 2.5]). No distinct differences, however, were found compared to extruded IVDs.

## 4. Discussion

In this study, we found that lumbar IVDs directly adjacent to IVD extrusions showed lower gagCEST values than IVDs non-adjacent to extrusions. Furthermore, lumbar IVDs of patients with nsLBP exhibited the lowest overall gagCEST values, lower than the controls and slightly lower than patients with radiculopathy.

Lumbar IVD extrusions are a known and common cause of LBP that not only lead to local pain, but frequently are accompanied by radicular pain, sensory deficits and/or muscle weakness due to the affection of nerve roots [34,35]. Pathophysiologically, proteoglycan depletion of IVDs results in tissue dehydration (of both AF and NP) that brings about decreases in IVD height. These changes are characteristic of LDDD and subsequently increases each IVD’s susceptibility to mechanical stress that predisposes to tears and fissures of the AF, and eventually results in IVD extrusions [36]. In the present study, patients who suffered from radiculopathy demonstrated IVD extrusions in their MRI scans. The extruded IVDs showed substantially lower gagCEST values than those of asymptomatic volunteers, which is not surprising since proteoglycan depletion corresponds well with the pathophysiologic concept of IVD degeneration preceding extrusion [37]. Interestingly, IVDs adjacent to the extruded IVD, i.e., directly above and/or below, also demonstrated lower gagCEST values than IVDs that were non-adjacent to the extruded IVD. It remains speculative whether the distinct proteoglycan depletion of the adjacent IVDs was caused by the extrusion and the subsequently altered biomechanics or whether it was simply altered due to exposure to the same mechanical stress that eventually led to IVD extrusion. In favor of the former, overall gagCEST values of patients with radiculopathy were similar to those of asymptomatic volunteers and did not demonstrate signs of advanced degeneration. Thus, one could argue that IVD extrusions affect the biomechanical integrity of the surrounding spine leading to advanced segmental degeneration that originates at the adjacent segments. In favor of this hypothesis, Masui et al. designed a longitudinal study and followed 21 patients over seven years. They found that progressive IVD degeneration regularly occurred after conservative treatment of symptomatic IVD extrusions [38]. Most of their patients showed no signs of morphologic IVD degeneration at baseline (morphologic MRI). Notably, compositional MRI techniques that may allow for the detection of premorphologic IVD changes were not performed in their study. Concurring findings were presented in a systematic review by Schroeder et al. [39], who also concluded that such changes were likely physiological. Keeping these preliminary findings in mind, it would be of interest to perspectively investigate the development of the progressive degeneration of adjacent segments following IVD extrusion in a longitudinal manner. This might contribute to a better understanding of the relationship between IVD extrusion, LBP, and LDDD. Eventually, this might facilitate the identification of “IVDs at risk” that in the future, if disease-modifying treatment is available by then, may influence patient care [40,41,42].

LDDD not only adds to the development of IVD extrusion, but by itself is considered a potential cause and contributor to chronic LBP [43,44,45,46], even though it might also occur in asymptomatic individuals [47]. Several studies underlined the high prevalence of degenerative spinal changes in the asymptomatic population [48,49]. Therefore, MR imaging should be avoided in the setting of acute and subacute LBP [50]. Our results show that patients with nsLBP had significantly lower gagCEST values than asymptomatic volunteers and somewhat lower values than patients with radiculopathy. These findings are well aligned with one of our previous studies that demonstrated patients with non-radicular LBP to show more severe signs of early IVD degeneration than asymptomatic references [19]. Therefore, the results of this study emphasize the potential association of LBP and LDDD [37], even in cases of premorphological degenerative changes of IVDs, as also suggested by previous studies [51,52]. Thus, compositional MRI could potentially be used in the primary diagnostic work-up of patients with LBP and in follow-up measurements after therapeutic intervention to evaluate treatment success. However, the limited correlation between morphologic changes and clinical outcome as shown in previous studies should be considered in this respect [53].

When interpreting our results, some limitations have to be considered.

First, despite including a sizable study population, each study cohort was relatively small. Therefore, our results have to be considered as preliminary and future studies are required to confirm our findings in larger clinical populations. Second, gagCEST imaging still requires histological validation in human subjects. However, for obvious ethical circumstances, our gagCEST data were not histologically correlated. Such validation remains to be performed directly, in human cadaveric studies, or indirectly, via IVD biopsy during surgery. Third, patients with nsLBP suffered from more severe GAG depletion and higher morphologic IVD degeneration (quantified by Pfirrmann grades ≥ 3) than asymptomatic volunteers. However, to mitigate this limitation, we statistically corrected for differences in Pfirrmann grading in the LMM. Fourth, we only performed one single compositional MRI technique, i.e., gagCEST, while other promising methods for the detection of premorphologic degenerative changes of IVDs, such as T2* mapping [54], were not assessed and remain to be studies. Fifth, the study population was rather heterogeneous, e.g., with a wide age range, which was partially mitigated by matching across the three cohorts. Sixth, gagCEST imaging is ideally performed at 7 T, and there is ongoing discussion as to whether it is feasible at 3 T MRI systems. In addition, it is worth noting that, to this day, gagCEST imaging lacks a standardized protocol as well as normative values and is highly dependent on the individual scanning system, overall hampering the comparability between different sites. To expand gagCEST imaging beyond feasibility studies, expert imaging recommendations as for other imaging biomarkers seem most valuable [55].

## 5. Conclusions

In conclusion, more advanced GAG depletion in nsLBP and in IVDs adjacent to IVD extrusions in radiculopathy indicates close interrelatedness of clinical pathology and compositional and structural IVD changes in lumbar spine degeneration. These findings underline the potential diagnostic value of non-invasive gagCEST imaging in (a) quantifying tissue composition and detecting premorphological IVD degeneration; (b) in differentiating patterns of IVD degeneration in common clinical disorders of the lumbar spine, and (c) in identifying “IVDs at risk”.

## Figures and Tables

**Table 1:** Demographic and clinical information of the study population.

	Patients with Non-Specific Low Back Pain	Patients with Radiculopathy	Volunteers
Cohort size (*n*)	16	18	20
Age (years)	59 ± 17.5	57.5 ± 22.5	54.5 ± 11.5
Gender female/male	10/6	10/8	11/9
IVD extrusion (*)	0	18 (one in each patient)	0
IVD protrusion	19	9	8
LBP (*n*)	16	18	0
Radicular pain (*n*)	0	18	0
Sensory deficit (*n*)	0	14	0
Muscle weakness (*n*)	0	0	0

Age data are presented as means ± standard deviations. For each study cohort, age, sex, the presence and distribution of IVD extrusions as well as the clinical presentation are given. Abbreviations: IVD —intervertebral disc. Detailed distribution of IVD extrusions: L1/2 (*n* = 0), L2/3 (*n* = 0), L3/4 (*n* = 0), L4/5 (*n* = 9), L5/S1 (*n* = 9). (*) IVD extrusion was defined as present when the base of the protruded disk material was narrower than its dome. IVD protrusion was defined as present when the base of the protruded disk was broader than its dome. LBP: low back pain.

**Table 2:** Detailed Magnetic Resonance Imaging (MRI) Sequence Parameters.

	Sequence					
	STIR	T2w TSE	T1w TSE	T2w TSE	CEST	WASSR
Imaging Plane	Sagittal	Sagittal	Sagittal	Transversal	Midsagittal	Midsagittal
TE (ms)	57	95	9.5	106	5.1	5.1
TR (ms)	3800	3500	650	5200	10	10
Flip Angle (°)	150	160	150	160	10	10
Slice Thickness (mm)	4	4	4	4	5	5
FoV (mm × mm)	300 × 300	300 × 300	300 × 300	190 × 190	300 × 300	300 × 300
Pixel Size (mm × mm)	0.8 × 0.8	0.7 × 0.7	0.7 × 0.7	0.6 × 0.6	1.6 × 1.6	1.6 × 1.6
Number of Slices	15	15	15	38	1	1

Imaging plane, echo time (TE), repetition time (TR), flip angle, slice thickness, field of view (FoV), pixel size, and number of slices are given for all sequences (Short Tau Inversion Recovery (STIR), T2-weighted turbo spin echo (T2w TSE), T1w TSE, chemical exchange saturation transfer (CEST) and water saturation shift referencing (WASSR) sequences).

**Table 3:** Mean imaging measures as a function of the study cohort, i.e., patients with radiculopathy, non-specific low back pain (nsLBP) and asymptomatic volunteers, and intervertebral disc (IVD) segment level, i.e., L1/2–L5/S1. Data are given as means [99% confidence intervals]. Glycosaminoglycan chemical exchange saturation transfer (gagCEST) values were compared using a linear mixed model comprising a subject-specific random intercept, while the Pfirrmann grades were compared using the Kruskal–Wallis test followed by Dunn’s post-hoc test wherever appropriate.

		Segment					
		L1/2	L2/3	L3/4	L4/5	L5/S1	Overall
gagCEST values (%)	Radiculopathy	2.5 [1.4; 3.5]	2.1 [1.3;2.8]	1.7 [1.0; 2.3]	1.2 [0.6; 1.9]	1.5 [0.5; 2.6]	1.8 [1.4; 2.1]
	nsLBP	1.3 [0.5; 2.1]	1.1 [0.5; 1.7]	1.2 [0.6; 1.8]	1.1 [0.5; 1.7]	2.3 [1.3; 3.3]	1.3 [1.0; 1.6]
	Volunteers	2.5 [1.7; 3.2]	2.0 [1.3; 2.5]	1.5 [1.0; 2.0]	1.7 [1.2; 2.2]	2.1 [1.2; 3.0]	1.9 [1.6; 2.2]
Pfirrmann grade (1–5)	Radiculopathy	2.3 [2.0; 2.7]	2.4 [2.0; 2.6]	2.6 [2.2; 2.9]	2.8 [2.5; 3.1]	2.9 [2.6; 3.2]	2.6 [2.4; 2.7]
	nsLBP	2.5 [2.1; 2.9]	2.7 [2.0; 2.7]	2.6 [2.2; 3.0]	2.8 [2.3; 3.2]	3.1 [2.6; 3.6]	2.7 [2.6; 2.9]
	Volunteers	2.2 [1.9; 2.5]	2.3 [2.0; 2.6]	2.4 [2.0; 2.7]	2.4 [2.2; 2.6]	2.5 [2.2; 2.8]	2.4 [2.2; 2.5]

**Table 4:** Comparative analysis of glycosaminoglycan chemical exchange saturation transfer (gagCEST) values of intervertebral discs (IVDs) adjacent to IVD extrusions and IVDs non-adjacent to IVD extrusions of patients with radiculopathy. Data are means [99% confidence intervals]. The mean gagCEST values were compared with a linear mixed model comprising a subject-specific random intercept.

gagCEST Values (%)	Cohort		Mean [99% CI]
	Patients with radiculopathy	IVDs adjacent to IVD extrusions	0.9% [0.3; 1.5]
		IVDs non-adjacent to IVD extrusions	2.1% [1.7; 2.5]
		Extruded IVDs	1.0% [0.2; 1.8]

## Data Availability

Data can be provided by the authors upon reasonable request.
